# Deep sequencing of hepatitis B virus basal core promoter and precore mutants in HBeAg-positive chronic hepatitis B patients

**DOI:** 10.1038/srep17950

**Published:** 2015-12-09

**Authors:** Linlin Yan, Henghui Zhang, Hui Ma, Di Liu, Wei Li, Yulin Kang, Ruifeng Yang, Jianghua Wang, Gaixia He, Xingwang Xie, Hao Wang, Lai Wei, Zuhong Lu, Qixiang Shao, Hongsong Chen

**Affiliations:** 1 Department of Immunology, and Jiangsu Key Laboratory of Medical Science and Laboratory Medicine, School of Medicine, Jiangsu University, Zhenjiang 212013, Jiangsu, China; 2 Peking University People’s Hospital, Peking University Hepatology Institute, Beijing Key Laboratory of Hepatitis C and Immunotherapy for Liver Diseases, Beijing, 100044, China; 3 Institute of Microbiology, Chinese Academy of Sciences, Beijing, 100101, China; 4 Department of Biomedical Engineering, College of Engineering, Peking University, Beijing, 100871, China

## Abstract

Mutants in the basal core promoter (BCP) and precore (PC) regions of hepatitis B virus (HBV) genome are associated with the progression of chronic hepatitis B (CHB) infection. However, quasispecies characteristics of naturally occurring mutants in those regions in HBeAg-positive CHB patients has not been well described, partly limited by quantitative assay. This study aimed to develop an Ion Torrent deep sequencing assay to determine BCP and PC mutant percentages in HBeAg-positive CHB patients who were treatment naïve and correlate them with different viral and host factors. Our results showed that Ion Torrent deep sequencing could achieve high accuracy (R^2^>0.99) within a dynamic range between 1% and 100%. Twelve hotspots with prevalence of greater than 20% were observed in EnhII/BCP/PC regions. G1719T, T1753V, A1762T and G1764A were genotype C related. BCP A1762T/G1764A double mutants were generally accompanied with PC 1896 wild type or lower PC G1896A mutant percentage. Lower serum HBeAg and HBsAg levels were associated with higher BCP A1762T/G1764A mutant percentages (≥50%). ALT levels were higher in patients with PC G1896A mutant percentage greater than 10%. In conclusion, deep sequencing such as Ion Torrent sequencing could accurately quantify HBV mutants for providing clinical relevant information during HBV infection.

Hepatitis B virus (HBV) is a 3.2 kb circular, partially double-stranded DNA virus, but replicates through reverse transcription of an RNA intermediate^1^. The lack of proofreading function causes a high mutation rate during chronic HBV infection^2^. Mutations in the basal core promoter (BCP) and precore (PC) regions occur most commonly, which reduce and abolish the production of HBeAg respectively^3^,^4^. The consequent decrease or lack of HBeAg production might trigger the immune escape of virus, which subsequently favoring the persistence of HBV infection.

Accumulated studies have shown the impact of BCP and PC mutants on the progression of chronic hepatitis B (CHB) and antiviral response. BCP A1762T/G1764A double mutants increase the risk of liver cirrhosis and hepatocellular carcinoma (HCC)^5^,^6^,^7^,^8^,^9^, PC G1896A mutant decreases the risk of HCC^6^, although the impact of PC G1896A mutant on HCC development remain controversial. In addition, pretreatment BCP/PC mutant percentages are associated with the response rate to interferon therapy^10^. Therefore, BCP and PC mutants as critical factors in the progression and treatment of CHB, early detection of these mutants with low frequencies is very necessary.

However, most previous studies analyzed HBV mutants using qualitative assay, such as direct sequencing, which limited the quantitative detection of mutants with low frequencies. Prior studies mostly only focused on certain specific mutants, which overlooked the potential influence of other variants. Furthermore, the study of the quasispecies characteristics of BCP and PC mutants in HBeAg-positive CHB patients was limited. Therefore, a highly sensitive method is urgently to be developed to analyze the percentages of HBV mutants.

With recent advances in next-generation sequencing (NGS) technologies such as Ion Torrent Personal Genome Machine (PGM) sequencing, quantification of viral mutants has become possible and more sensitive. Ion Torrent PGM sequencing is based on semiconductor technology, which allowing for low-cost, high sensitivity, large-scale production and high throughput^11^. It has been broadly applied in determining microbial community diversity and cancer unique mutations^12^,^13^. In this study, we developed a fast and sensitive quantitative method to analyze HBV mutants based on Ion Torrent PGM sequencer platform. We then took advantage of this assay to analyze the percentages of HBV EnhII/BCP/PC mutants in 58 HBeAg-positive CHB patients and correlate them with different viral and host factors.

## Results

### Development and validation of Ion Torrent PGM sequencer platform for quantification of BCP and PC mutants

**Figure 1:**
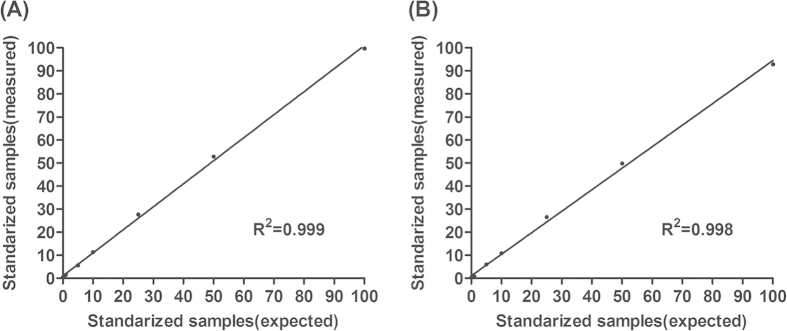
Validation of Ion Torrent PGM sequencing for quantification of BCP and PC mutants. Validation of Ion Torrent PGM sequencing for quantification of (**A**) BCP and (**B**) PC mutants using reference plasmids with a range of pre-defined mutant ratios, 0%, 0.1%, 0.5%, 1%, 5%, 10%, 25%, 50% and 100%. Each sample was measured in triplicate and data presented as mean  ±  SE.

To confirm the accuracy of Ion Torrent PGM sequencing for quantification of HBV mutants, two reference plasmids containing A1762/G1764/G1896 and T1762/A1764/A1896 were constructed, which serve as BCP/PC wild type and mutant type plasmids respectively. Then, we mixed the BCP/PC mutant type and wild type plasmids as the following mutant ratios, 0%, 0.1%, 0.5%, 1%, 5%, 10%, 25%, 50% and 100%. Each sample was amplified and sequenced in triplicate. Clone sequencing was performed meanwhile. Our results showed that the measured percentages of BCP/PC mutants were quite similar to the expected mutant percentages, with R^2^ of 0.99 (Fig. 1A,B). The difference between clone sequencing and Ion Torrent sequencing can be found as Supplementary Fig. S1. The standard error (SE) of triplicate measurement of each sample was within 1%. These results indicated that Ion Torrent PGM sequencing could achieve high accuracy and reproducibility within a dynamic range between 1% and 100%. Therefore, we further applied this assay to analyze the percentages of HBV EnhII/BCP/PC mutants in 58 HBeAg-positive CHB patients who were all treatment naïve.

### Characteristics of the study cohort

Table 1 showed the demographic data of 58 HBeAg-positive CHB patients. The majority (79.3%) of the study participants were male. The prevalence of genotype C infection was 69.0%.

### Prevalence of mutants in the EnhII/BCP/PC regions

**Figure 2:**
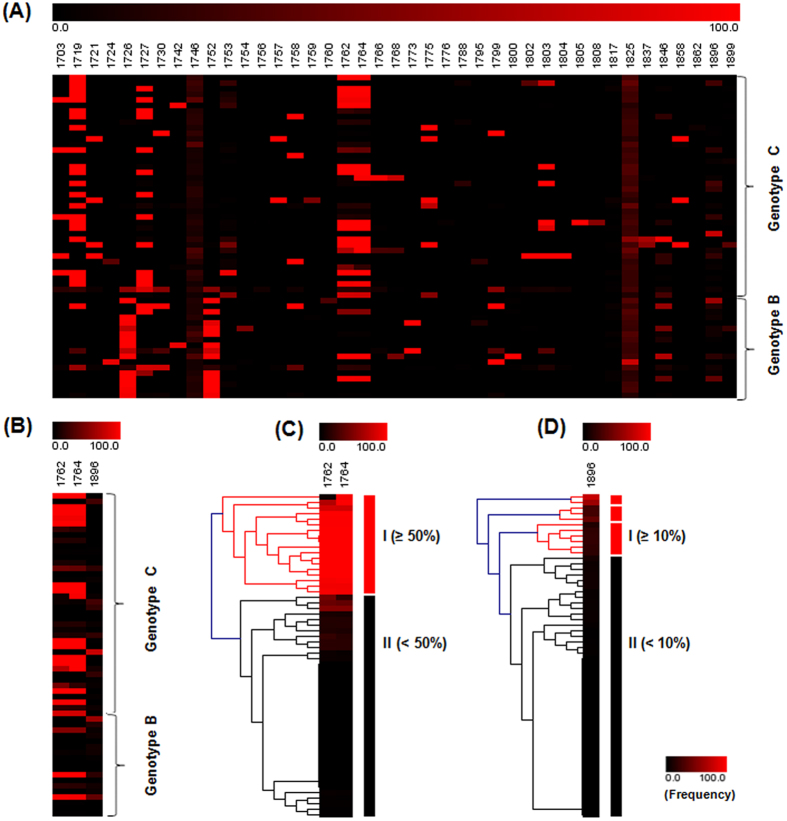
Mutation spectrum and hierarchical clustering analysis of BCP and PC mutants. (**A**) Quasispecies spectrum of mutants in the EnhII/BCP/PC regions in 58 HBeAg-positive CHB patients. (**B**) Combinational patterns of BCP A1762T/G1764A and PC G1896A mutants. Hierarchical clustering analysis of (**C**) BCP A1762T/G1764A and (**D**) PC G1896A mutants.

Overall, 41 SNPs were detected in EnhII/BCP/PC regions. The distribution and percentages of these mutants among 58 HBeAg-positive CHB patients were shown in Fig. 2A. Most SNPs dispersed distribution. Twelve hotspots that had mutant types with prevalence of greater than 20% were observed at positions nt.1719, nt.1726, nt.1727, nt.1746, nt.1752, nt.1753, nt.1762, nt.1764, nt.1817, nt.1825, nt.1846, and nt.1896 (Table 2). The prevalence of nt.1746, nt.1762/1764, nt.1825, nt.1896 mutant reached 96.6%, 60.3%, 100% and 50%, respectively (Table 2). Genotype C infection had a higher prevalence of BCP A1762T/G1764A mutants than genotype B infection (70.0% vs 38.9%, *P* = 0.0413) (Table 2), which is coincident with most previous studies. No significant difference was observed for PC G1896A mutant (45.0% vs 61.1%, *P* = 0.3950). In addition, among the twelve hotspots, G1719T and T1753V were significantly associated with genotype C (*P* < 0.05), while A1726C and A1752G/T were genotype B related (*P* < 0.05) (Table 2). And we compared CHB patients with that of hepatocellular carcinoma patients, the results are summarized in supplementary Table S2 and Fig. S2.

### HBV BCP A1762T/G1764A and PC G1896A combinational patterns

Of all the above mutants in BCP and PC regions, we defined the A1762T/G1764A double mutants as BCP mutants and G1896A as PC mutant. The mean percentage of BCP and PC mutants were 54.1 ± 42.9/56.2 ± 44.0 and 13.9 ± 17.9, respectively (Table 3). Genotype C infection, compared to genotype B infection, had similar PC mutant percentage (13.3 ± 18.2 vs 14.9 ± 18.3, *P* = 0.8154) and higher BCP mutant percentages, but without statistical significance (58.1 ± 42.5/60.8 ± 43.6 vs 38.2 ± 44.2/37.8 ± 44.0, *P* > 0.05) (Table 3). In this study cohort, 32.8% of patients had BCP mutants only, 22.4% had PC mutant only and 27.6% had both BCP and PC mutants (Table 3). BCP mutants were generally accompanied with PC wild type (69.3 ± 42.8/68.8  ±  43.3/0) or lower PC mutant percentage (36.1 ± 36.7/41.3 ± 41.4 vs 10.3 ± 10.4, *P*  <  0.05) (Fig. 2B and Table 3). And the ratios and combinations of quasispecies can be found as Supplementary Fig. S3.

### Correlation between BCP/PC mutants and different viral and host factors

To determine the correlation between BCP/PC mutants and different viral and host factors, two clusters of BCP and PC mutants were separately generated using hierarchical clustering analysis, and the dividing point was 50% and 10%, respectively (Fig. 2C,D). We then compared the viral and host factors between different clusters. As shown in Table 4, BCP wild type or BCP mutants (<50%) were more prevalent in genotype B infection than in genotype C infection (*P* = 0.0341). Serum HBeAg and HBsAg levels were significantly lower in patients with higher BCP mutant percentages (≥50%) compared to those with BCP wild type or lower BCP mutant percentages (<50%) (*P* =  0.0127, *P* =  0.0183). Patients with PC mutant (≥10%) had higher ALT levels than those with PC wild type or with lower PC mutant percentage (<10%) (*P* = 0.0436). No significant correlation was abserved between BCP/PC mutants and viral loads. In addition, PC wild type or PC mutant (<10%) was more prevalent in male patients (*P* =  0.0387). Besides, the impacts of the ratios of A1762/T1762 or G1764/A1764 on viral load and HBeAg / HBsAg levels were also analyzed (see Supplementary Fig. S4).

### Amino acid transitions induced by mutations in the EnhII/BCP/PC regions

**Figure 3:**
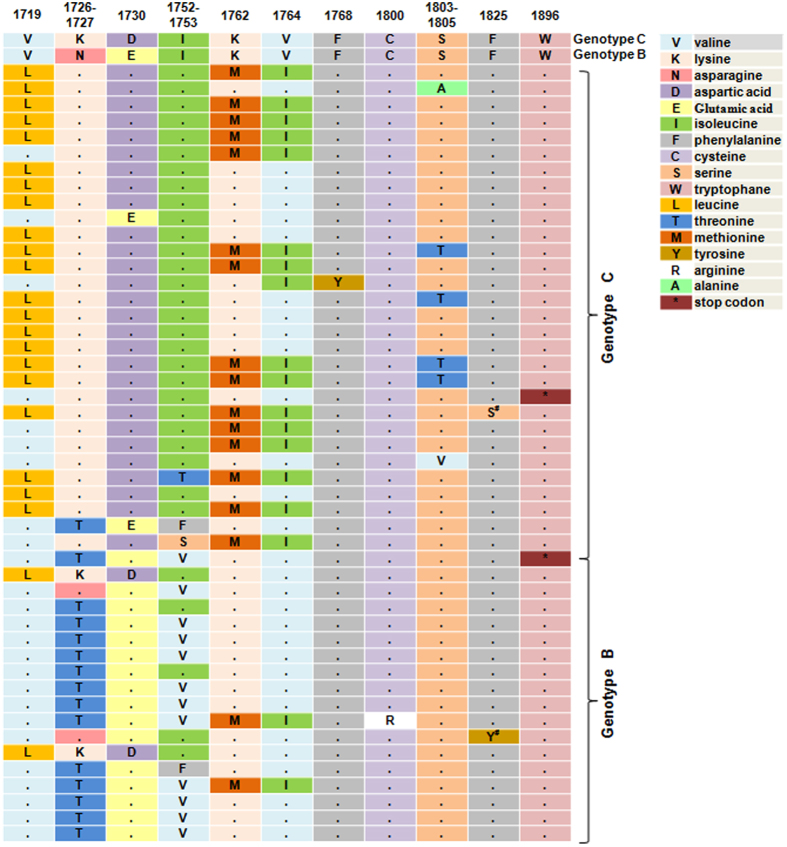
Amino acid transitions induced by mutations in the EnhII/BCP/PC regions. S*: T1825C affects codon 151 of the X gene (F151S); Y*: T1825A affects codon 151 of the X gene (F151Y) and codon 4 of the PC gene (F4L).

We then translated nucleotide sequences to corresponding peptide sequences. Only SNPs that had mutant types with percentage of greater than 50% were considered here. As shown in Fig. 3, besides classical A1762T/G1764A and G1896A mutants affecting codons 130/131 of X gene (K130M/V131I) and codon 28 of PC gene (W28stop), another 12 SNPs (nt.1719, nt.1726, nt.1727, nt.1730, nt.1752, nt.1753, nt.1768, nt.1800, nt.1803, nt.1804, nt.1805 and nt.1825) also caused amino acid changes. Amino acid transitions induced by G1719T, A1726C, A1752G/T and BCP A1762T/G1764A mutants had higher prevalence of 39.7% (23/58), 29.3% (17/58), 27.6% (16/58) and 31.0% (18/58), which mainly occurred in genotype C (21/23), genotype B (16/17), genotype B (13/16) and genotype C (16/18) infection patients, respectively.

## Discussion

Several studies have demonstrated the impact of BCP and PC mutants on the progression of chronic HBV infection^5^,^6^,^7^,^8^,^9^,^14^,^15^,^16^. However, most previous studies did not particularly categorise CHB patients into HBeAg-positive and HBeAg-negative and analyzed these mutants using qualitative assay. In this study, we developed an Ion Torrent PGM sequencing based quantitative assay to analyze the percentages of HBV mutants, which achieved high accuracy (R^2^>0.99) within a dynamic range between 1% and 100% and excluded the influence of PCR bias. Ion 318 chip could generate 1Gb pairs (Gbp) of sequence data, with the average coverage more than 50,000 reads per sample, which allows for the detection of mutants with low frequencies. The primer barcode recognition design grants Ion Torrent sequencing the ability of parallel sequencing up to 26 samples per chip, greatly reducing the personal cost and turnaround time. Thus, we could conclude that deep sequencing such as Ion Torrent sequencing is a reliable platform for quantification of HBV mutants.

In addition to BCP A1762T/G1764A and PC G1896A mutants, high prevalence of mutants were also observed at nt.1719, nt.1726, nt.1727, nt.1746, nt.1752, nt.1753, nt.1817, nt.1825 and nt.1846. A previous study has shown that G1719T, A1726C, A1727T, T1753V and A1846T were associated with advanced liver diseases^17^,^18^, indicating that other mutants other than A1762T/G1764A/G1896A should be took into account in evaluating the progressive liver diseases in the future. Meanwhile, we found that A1762T, G1764A, G1719T and T1753V were significantly associated with genotype C, while A1726C and A1752G/T were genotype B related. Genotype C infection is more likely to progress to HCC^19^,^20^ and is associated with a lower response rate to interferon treatment compared to genotype B infection^21^. Thus, we speculated that these genotype related mutants might play specific roles in the progression of liver diseases and antiviral response.

In this study, we found that BCP mutants were generally accompanied with PC wild type or lower PC mutant percentage. Several studies revealed that BCP mutants are risk factors of cirrhosis and HCC^5^,^6^,^7^,^8^,^9^, PC mutant decreases the risk of HCC and was suggested to possess a protective effect against liver lesions^6^,^22^. However, there are also some discrepant findings about the impact of PC mutant on the development of HCC. A retrospective study from taiwan revealed that pretreatment PC mutant percentage was positively related with interferon induced HBeAg seroconversion^10^, but another study suggested that pretreatment PC wild type is more responsive to IFN-alpha^23^. Therefore, this pattern of BCP mutants combined with PC wild type or lower PC mutant percentage might have several possible impacts during chronic HBV infection. The exact role of this combinational pattern would be further observed in the later long-term follow-up study.

The BCP and PC mutants not only alter the expression of HBeAg, but also affect viral replication^24^. Studies of the impact of BCP and PC mutants on viral replication remain controversial: no effect^25^,^26^, increase viral replication^24^,^27^ or reduce viral replication^28^. A recent study suggested that BCP mutants are associated with lower viral loads in HBeAg positive individuals, PC stop mutation is not associated with viral loads^29^. However, no significant correlation was observed between BCP mutants and viral loads in this study. It is noted that, all patients enrolled in this study were HBeAg-positive immune clearance phase CHB patients. HBeAg seroconversion occurred during immune clearance phase, which is often accompanied by the reduction of HBV replication^30^. But the decline of serum viral load generally occurred within 1 year before HBeAg seroconversion^31^. Therefore, the time of measurement might influence the view of the effect of BCP mutants on viral replication. It is known that PC stop mutant abolishs the secretion of HBeAg^4^, but no significant correlation was found between PC mutant and HBeAg reduction in this study, possibly because the PC mutant percentage (13.9 ± 17.9) is not high enough to significantly reduce the total HBeAg levels. Furthermore, we found that lower HBsAg level was associated with higher BCP mutants. BCP mutants were reported to be associated with higher chance of HBeAg seroconversion^10^,^32^. Sustained HBeAg seroconversion favors the occurring of HBsAg seroclearance, a state closest to “cure” of CHB^33^. Thus, this finding promoted a hypothesis that whether BCP mutants are associated with HBsAg loss.

We developed a sensitive deep sequencing based assay to quantify HBV mutants, which overcame the deficiencies associated with quantification, cost, runtime and large-scale production. We enrolled HBeAg-positive CHB patients who were all treatment naïve, ensuring that the mutations are naturally occurring during chronic HBV infection. But, the small sample size might compromise our conclusion to some extent and hampered a further analysis of the 41SNPs. Therefore, further studies are required.

In conclusion, by Ion Torrent PGM sequencing, we described the quasispecies characteristics of HBV mutants in EnhII/BCP/PC regions in 58 HBeAg-positive CHB patients and determined the correlation between these mutants and different viral and host factors. This study provided a fast and sensitive quantitative platform for screening HBV mutants during the long-term HBV infection period.

## Materials and Methods

### Patients

This study included 58 HBeAg-positive CHB patients. The included patients met the following criteria: 18–70 years old, positive HBsAg for at least 6 months, HBeAg positive, serum ALT levels over 2–10 times the upper limit of normal (ULN, 40U/L), HBV DNA > 1 × 10^5^ copies/ml, white blood cell (WBC) > 3.0 × 10^9^/L, granulocyte > 1.5 × 10^9^/L, platelet > 100 × 10^9^/L, and urine pregnancy test negative. Patients with any causes of liver diseases other than CHB, pregnant and/or breast-feeding women, individuals received immune regulator or antiviral treatment within previous 6 months before the commencement of this study, patients with compensated or decompensated cirrhosis, anti-human immunodeficiency virus (HIV) positive, and those with a history of renal dialysis or organ transplantation were excluded. This study was conducted in accordance with the ethics principles of the Declaration of Helsinki and was approved by the Ethics Committee of Peking University People’s Hospital. All patients signed written informed consents.

### Extraction of serum HBV DNA

HBV DNA was extracted from 200 μl serum samples using QIAamp DNA Blood Mini kit (Qiagen, Germany) and eluted into 50 μl buffer AE according to the manufacturer’s instructions. HBV genotype analysis was performed by direct sequencing.

### Laboratory Tests

Serum HBV DNA level was determined using the Cobas Taqman assay (detection limit, 12 IU/mL; Roche, Rotkreuz, Germany). Serum HBsAg level was quantified on the Architect i2000 (Abbott Laboratories, Abbott Park, IL, USA). The dynamic range is from 0.05 to 250 IU/mL. If the HBsAg level was higher than 250.0 IU/mL, the samples were 1:100 serially diluted to obtain a value falling within the dynamic range. Serum HBeAg quantification was performed using a home-brewed method as previously described^34^.

### Quantitative analysis of BCP and PC mutants by Ion Torrent PGM sequencing

The fragment of nucleotides 1678 to 1948 (including EnhII (nt.1685–1773), BCP (nt.1742–1849) and PC (nt.1814–1900)) of HBV genome was amplified by nested PCR using the AmpliTaq Gold^®^360 Master Mix (Applied Biosystems, Foster, CA, USA). First-round PCR was performed in a 20 μl reaction using primers B935 (nt.1240–1260, 5′-GAAGGTTTGTGGCTCCTCTG-3′) and MDC1 (nt.2304–2324, 5′-TTGATAAGATAGGGGCATTTG-3′)^29^. The final concentration of each primer was 0.5 μmol/L. PCR conditions consisted of 5 min hot start, 40 cycles of 95 °C for 30 s, 55 °C for 30 s, and 72 °C for 90 s, followed by a long extension of 7 min at 72 °C. Second-round PCR was carried out on 2.5 μl of the first round product in a 50μl reaction mixture. Because Ion 318 chip could run up to 26 samples per chip, 26 second-round PCR forward primers and 1 second-round PCR reverse primer were separately designed with unique barcodes (see supplementary Table S1). PCR conditions consisted of 5 min hot start, 40 cycles of 95 °C for 30 s, 59 °C for 30 s, and 72 °C for 90 s, followed by a long extension of 7 min at 72 °C. Nuclease-free H_2_O was used as negative control in nested PCR. The second-round products were confirmed by 2% agarose gel electrophoresis and purified using the QIAquick^®^ Gel Extraction Kit (Qiagen, Germany). Purified products were quantified on the Agilent Bioanalyzer^TM^ 2100 instrument (Agilent Technologies, Santa Clara, CA, USA) to analyze the size distribution and determine the molar concentration. Then pooling the amplicon libraries in equimolar concentrations for the downstream template preparation with the Ion PGM™200 Xpress™ Template Kit (Life Technologies, USA). The complete templates were loaded on the PGM™ System and sequenced using the Ion PGM™200 Sequencing Kit (Life Technologies, USA). In addition, two plasmids containing A1762/G1764/G1896 (wild type) and T1762/A1764/A1896 (mutant type) were used as quality control to ensure consistency in quantification in different runs.

### Sequence analysis

Ion Torrent PGM sequencer generated more than 50,000 reads, with the average length of more than 300 bp per sample, which encompassed the EnhII, BCP and PC regions (see supplementary Fig. S5). FASTQ sequence files were aligned with HBV genotype B or genotype C reference sequence derived from NCBI database using Burrows-Wheeler Alignment Tool (BWA-SW, version: 0.7.5a-r405). Samtools (version: 0.1.19 44428cd), a SNP calling software, was used to detect single nucleotide polymorphisms (SNPs). The corresponding peptide sequences were analyzed using Transeq software (version: EMBOSS: 6.6.0.0).

### Statistical analysis

Continuous variables were presented as mean  ±  standard deviation (SD) and categorical variables were presented as frequencies (percentages). Student’s t-test, χ^2^ test and Fisher’s exact test were performed to analyze data where appropriate. Linear regression analysis was performed to identify the fitting degree between measured percentages and true percentages.

Heat map and hierarchical clustering analysis were performed to show the distribution and percentages of mutants using Genesis software (version 1.7.6, Graz, Austria). Statistical analyses were conducted using the SPSS statistical software (version 17.0, Chicago, IL, USA). P < 0.05 was considered as statistically significant.

## Additional Information

**How to cite this article**: Yan, L. *et al.* Deep sequencing of hepatitis B virus basal core promoter and precore mutants in HBeAg-positive chronic hepatitis B patients. *Sci. Rep.* **5**, 17950; doi: 10.1038/srep17950 (2015).

## Supplementary Material

Supplementary Information

## Figures and Tables

**Table 1:** Characteristics of 58 HBeAg-positive CHB patients

Characteristics	CHB (n = 58)
Age (years)	30.4 ± 9.9
Sex	
Female	12 (20.7)
Male	46 (79.3)
HBV genotype	
B	18 (31.0)
C	40 (69.0)
Anti-HBe	
Positive	0 (0.0)
Negative	58 (100.0)
Serum HBV-DNA levels (copies/ml)	
10^5^–10^6^	3(5.2)
10^6^–10^9^	39 (67.2)
≥10^9^	16 (27.6)
Serum HBsAg levels (IU/mL)	
100–999	1 (1.7)
1,000–10,000	19 (32.8)
≥10,000	38 (65.5)
ALT(U/L)	175.5 ± 87.4
AST(U/L)	93.2 ± 62.6

CHB, chronic hepatitis B; HBV, hepatitis B virus; HBeAg, hepatitis B e antigen; anti-HBe, antibodies against HBeAg; HBsAg, hepatitis B surface antigen; ALT, alanine transaminase; AST, aspartate transaminase. Data presented as mean ± SD or no. (%).

**Table 2:** Sequence variants in the EnhII/BCP/PC regions of HBeAg-positive CHB patients

		Total	Genotype C	Genotype B	
Region	Variant	(n = 58) (%)	(n = 40) (%)	(n = 18) (%)	*P*value
EnhII	G1719T	28 (48.3)	24 (60.0)	4 (22.2)	0.0107
(1685–1773)	A1726C	18 (31.0)	3 (7.5)	15 (83.3)	<.0001
	A1727V^c^ (T1727V^b^)	30 (51.7)	24 (60.0)	6 (33.3)	
BCP	G1746A	56 (96.6)	39 (97.5)	17 (94.4)	0.5281
(1742–1849)	A1752G/T	17 (29.3)	3 (7.5)	14 (77.8)	<.0001
	T1753V	22 (37.9)	20 (50.0)	2 (11.1)	0.0075
	A1762T	35 (60.3)	28 (70.0)	7 (38.9)	0.0413
	G1764A	35 (60.3)	28 (70.0)	7 (38.9)	0.0413
PC	C1817G/A	18 (31.0)	11 (27.5)	7 (38.9)	0.5404
(1814–1900)	T1825C/A	58 (100.0)	40 (100.0)	18 (100.0)	
	A1846T	19 (32.8)	10 (25.0)	9 (50.0)	0.0757
	G1896A	29 (50.0)	18 (45.0)	11 (61.1)	0.3950

For all nucleotide positions in EnhII/BCP/PC regions, the most prevalent nucleotide type among HBV genotype B or genotype C infection patients in china were used as reference nucleotide. EnhII, enhancer II; BCP, basal core promoter; PC, precore; ^c^Genotype C; ^b^Genotype B; A, adenine; G, guanine; C, cytosine; T, thymine. Data presented as no. (%). *P*, Fisher’s exact test.

**Table 3:** Prevalence of BCP A1762T/G1764A and PC G1896A mutants

HBV characteristics	No. (%)	mean ± SD	*P*value
PC			
W(G1896)	29 (50.0)	0	
M (A1896)	29 (50.0)	13.9 ± 17.9	
Genotype C	18 (62.1)	13.3 ± 18.2	*0.8154
Genotype B	11 (37.9)	14.9 ± 18.3	
BCP			
W (A1762/G1764)	23 (39.7)	0	
M (T1762/A1764)	35 (60.3)	54.1 ± 42.9/56.2 ± 44.0	
Genotype C	29 (80.6)	58.1 ± 42.5/60.8 ± 43.6	*>0.05
Genotype B	7 (19.4)	38.2 ± 44.2/37.8 ± 44.0	
Combinations (1762/1764/1896)			
W/W/W (A/G/G)	10 (17.2)	0/0/0	
W/W/M (A/G/A)	13 (22.4)	0/0/18.4 ± 24.0	
M/M/W (T/A/G)	19 (32.8)	69.3 ± 42.8/68.8 ± 43.3/0	
M/M/M (T/A/A)	16 (27.6)	36.1 ± 36.7/41.3 ± 41.4/10.3 ± 10.4	<0.05

^*^T- test of mutant percentages between genotype C and genotype B. W: wild type; M: mutant type

**Table 4:** Comparison of viral and host factors between different BCP and PC clusters.

Characteristics	BCP (1762/1764) Clusters		*P*value	PC (1896) Clusters		*P*value
	I (n = 18)	II (n = 40)		I (n = 11)	II (n = 47)	
Age (years)	32.8 ± 9.7	29.4 ± 9.9	0.2280	32.6 ± 11.80	29.9 ± 9.4	0.4156
Sex						
Female	3 (25.0)	9 (75.0)	0.7361	5 (41.7)	7 (58.3)	0.0387
Male	15 (32.6)	31 (67.4)		6 (13.0)	40 (87.0)	
Genotype						
B	2 (11.1)	16 (88.9)	0.0341	4 (22.2)	14 (77.8)	0.7244
C	16 (40.0)	24 (60.0)		7 (17.5)	33 (82.5)	
HBV DNA levels (log_10_copies/ml)	8.3 ± 1.1	8.3 ± 1.1	0.9623	8.4 ± 1.0	8.3 ± 1.1	0.8884
HBeAg levels (PEI-U/mL)	372.7 ± 431.0	1376.0 ± 1619.0	0.0127	1117.0 ± 1863.0	1052.0 ± 1345.0	0.8938
HBsAg levels (log_10_ IU/mL)	4.0 ± 0.6	4.4 ± 0.6	0.0183	4.2 ± 0.6	4.3 ± 0.6	0.6830
ALT (U/L)	193.1 ± 101.2	167.6 ± 80.5	0.3079	223.1 ± 101.6	164.3 ± 80.9	0.0436

*P*, T- test. Data presented as mean ± SD or no. (%).
