# Supplementary material for: Deep sequencing of hepatitis B virus basal core promoter and precore mutants in HBeAg-positive chronic hepatitis B patients
Source: Sci Rep. 2015 Dec 9;5:17950. doi: 10.1038/srep17950 (PMC4673698; doi:10.1038/srep17950)
Supplement: Supplementary Information [file srep17950-s1.pdf]

Supplementary information

Deep sequencing of hepatitis B virus basal core promoter and precore mutants in HBeAg-positive chronic hepatitis B patients

Linlin Yan[1,2,*], Henghui Zhang[2,*], Hui Ma[2], Di Liu[3], Wei Li[3], Yulin Kang[4], Ruifeng Yang[2], Jianghua Wang[2], Gaixia He[2], Xingwang Xie[2], Hao Wang[2], Lai Wei[2], Zuhong Lu[4], Qixiang Shao[1], Hongsong Chen[2]

To whom correspondence should be addressed. Email: chenhongsong2999@163.com.

* These authors contributed equally to this work.

Supplementary Figures

Figure S1 The measurement of the accuracy of Ion Torrent PGM sequencing.

Figure S2 Sequence variants in the EnhII/BCP/PC regions of hepatitis B virus in HCC patients.

Figure S3 Combination patterns of BCP and PC mutations.

Figure S4 The HBV DNA/HBeAg/HBsAg levels and A1762/T1762 ratios in HBeAg positive CHB patients.

Figure S5 Illustration of the coverage and depths.

Supplementary Tables

Table S1 The list of primers utilized in nested PCR.

Table S2 Variants comparison of HBeAg positive CHB patients with that of HCC patients.

Supplementary figures

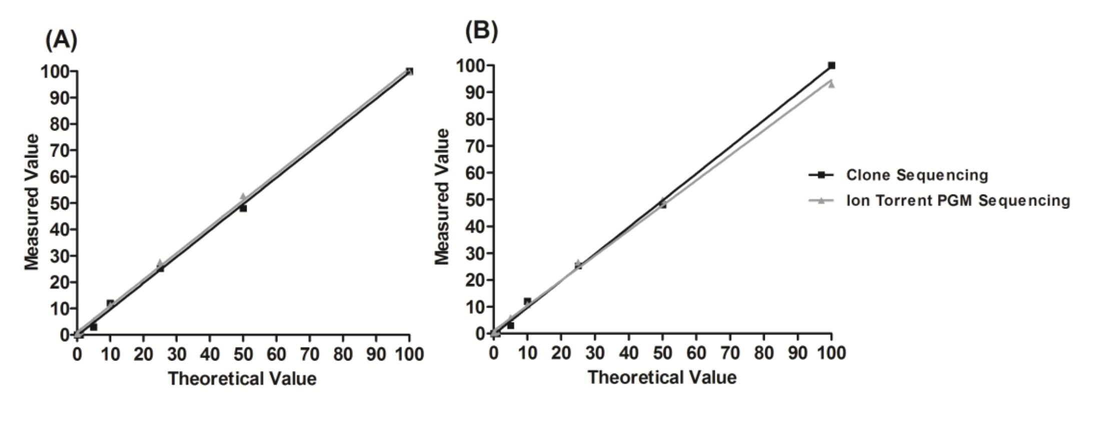

Figure S1 The measurement of the accuracy of Ion Torrent PGM sequencing. Standardized samples with a range of mutant ratios, 0%, 1%, 5%, 10%, 25%, 50% and 100% were used for clone sequencing. The BCP (A) and PC (B) mutant ratios detected by clone sequencing were compared with that by Ion Torrent PGM sequencing.

Clone sequencing was performed to provide a direct measurement of the accuracy of Ion Torrent deep sequencing. Figure S1 showed the difference of mutant ratios measured by clone sequencing and Ion Torrent sequencing. The results showed that the mutant ratios measured by Ion Torrent sequencing were highly consisted with that by clone sequencing.

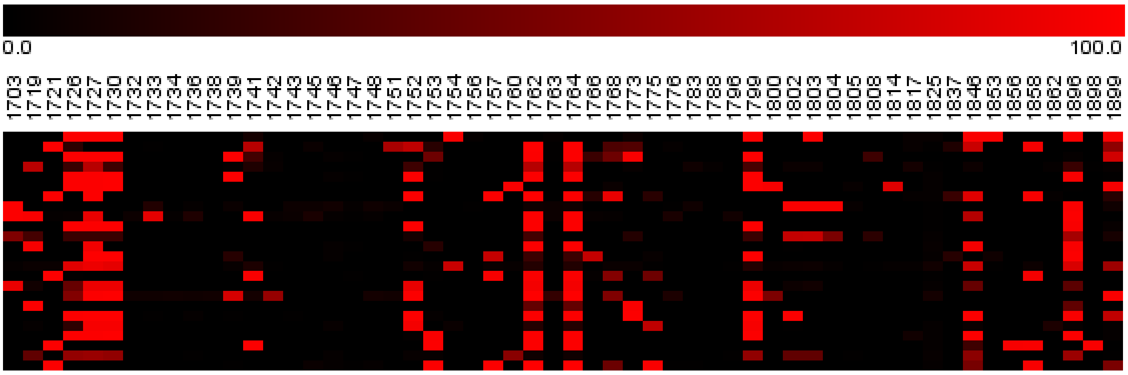

Figure S2 Sequence variants in the EnhII/BCP/PC regions of hepatitis B virus in HCC patients. The Distribution and percentages of mutations in the EnhII/BCP/PC regions of HBV genome among 24 HCC patients.

To compare CHB patients with that of hepatocellular carcinoma patients, we enrolled 24 hepatitis B associated hepatocellular carcinoma patients and performed Ion Torrent PGM sequencing. As shown in Fig.S2, 56 SNPs were detected in EnhII/BCP/PC regions of HBV. SNPs that had variant types with prevalence of greater than 50% were observed at positions 1726, 1727, 1730, 1752, 1753, 1762, 1764, 1799, 1825, 1846, 1896 and 1899 (Table S2). Twelve SNPs in the EnhII/BCP /PC region, 1721, 1726, 1730, 1752, 1762, 1764, 1768, 1773, 1799, 1802, 1846 and 1899, were higher prevalent in HCC patients than in CHB patients (P<0.05) (Table S2). Furthermore, the mean percentage of G1896A was higher in HCC patients than that in CHB patients, with statistical significance (66.60±9.18 VS 13.92±3.33, P < 0.0001).

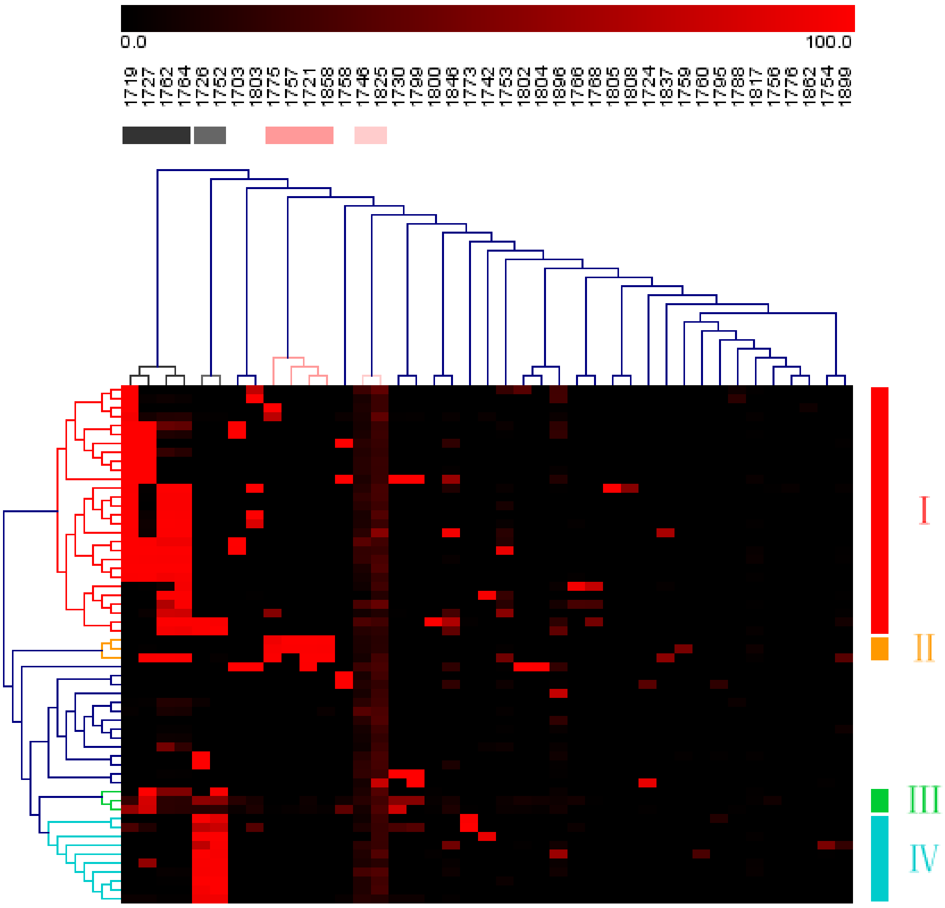

Figure S3 Combination patterns of BCP and PC mutations. Four types of combination patterns, I, II, III and IV, were generated through hierarchical clustering analysis on mutations and 58 HBeAg positive CHB patients simultaneously.

We performed hierarchical clustering on SNPs and patients simultaneously, hoping to find certain combinations of quasispecies. However, no obvious combinations of quasispecies were observed as shown in Fig.S3. Group I mainly contains the following patterns: 1719, 1719+1727, 1719+1762/1764, 1719+1727+1762/1764, 1762/1764, 1726+1752+1762/1764. Group II: 1775+1757+1721+1858; Group III: 1727+1762/1764+1726+1752; Group IV: 1726+1752.

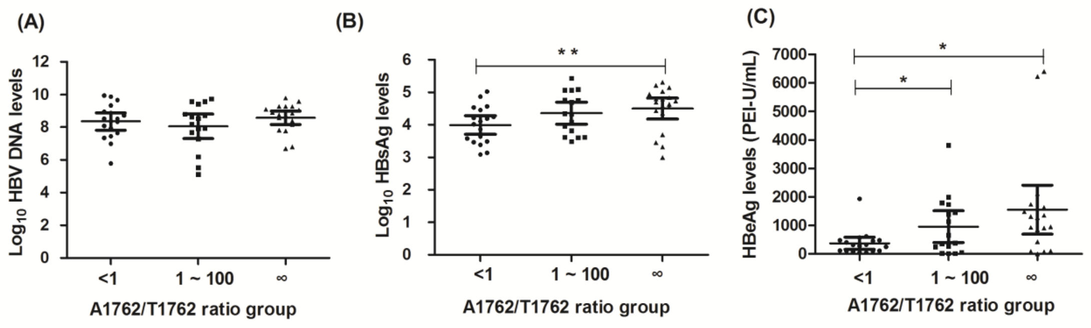

Figure S4 The HBV DNA/HBeAg/HBsAg levels and A1762/T1762 ratios in HBeAg positive CHB patients. The HBV DNA (A) and HBsAg (B) levels transformed by $\log_{10}$ and HBeAg (C) levels were plotted against different A1762/T1762 ratio groups. The horizontal lines indicate the mean (the middle long horizontal line) and the 95% confidence interval (the short horizontal lines above and below the mean).

In order to determine whether the ratios of A1762/T1762 or G1764/A1764 affects the viral load and HBeAg/HBsAg levels, we calculated the ratios of A1762/T1762 (which were consist with G1764/A1764) respectively and divided patients into three group according to the ratios. The results showed that no significant difference was existed between viral load and ratios of A1762/T1762 (Fig. S4 A). Higher ratios of A1762/T1762 could significantly increase the HBsAg and HBeAg levels (Fig. S4 B and C), but linear relation was not observed limited by small sample size. Thus, it is hardly to predict the viral load and HBeAg / HBsAg levels based on the deep sequencing results currently.

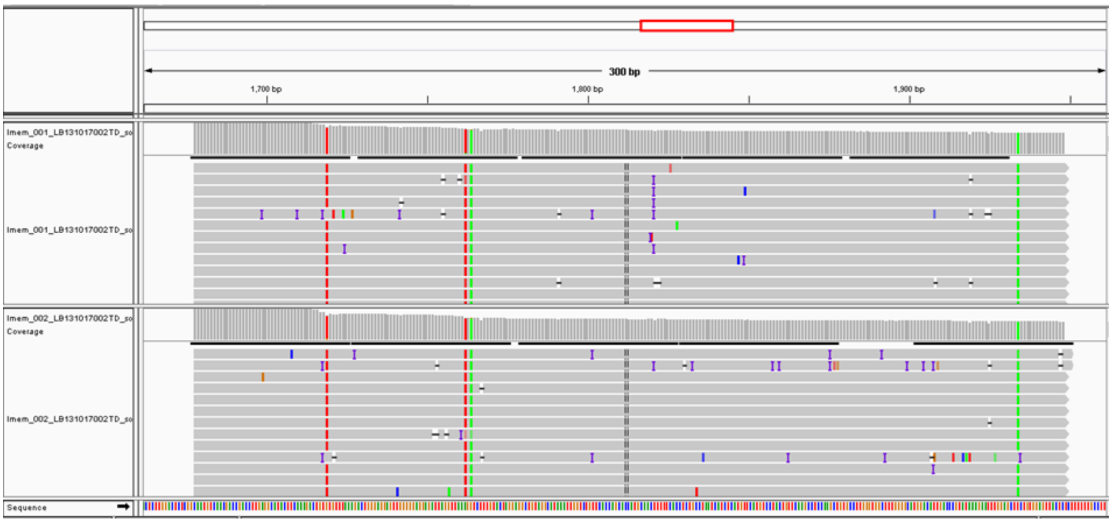

Figure S5 Illustration of the coverage and depths. This is reads mapping figure of two samples as example to show coverage and depth. Coverage for target region (271 bp) is 100% with more than 50,000 depths for each base pair.

Ion 318 chip could achieve a unidirectional sequence length of more than 400bp, without fracturing segments. In the practice, Ion Torrent PGM sequencer generated more than 50,000 reads, with the average length of more than 300bp per sample, which encompassed the EnhII / BCP / PC regions. As shown in Fig.S5, this is reads mapping figure of two samples as example to show coverage and depths. Coverage for target region (271 bp) is 100% with more than 50,000 depths for each base pair.

Supplementary Tables

Table S1 The list of primers utilized in nested PCR

Primer name	Primer sequence	Region
CPRF1	5’- CAATGTCAACGACCGACC -3’	1678–1695
CPRR1	5’- GAGTAACTCCACAGTAGCTCC -3’	1928–1948
Barcode 1-A-CPRF1	5’- CCATCTCATCCCTGCGTGTCTCCGACTCAG**CTAAGGTAA**CGATCAATGTCAACGACCGACC -3’	1678–1695
Barcode 2-A-CPRF1	5’- CCATCTCATCCCTGCGTGTCTCCGACTCAG**TAAGGAGAA**CGATCAATGTCAACGACCGACC -3’	1678–1695
Barcode 3-A-CPRF1	5’- CCATCTCATCCCTGCGTGTCTCCGACTCAG**AAGAGGATT**CGATCAATGTCAACGACCGACC -3’	1678–1695
Barcode 4-A-CPRF1	5’- CCATCTCATCCCTGCGTGTCTCCGACTCAG**TACCAAGAT**CGATCAATGTCAACGACCGACC -3’	1678–1695
Barcode 5-A-CPRF1	5’- CCATCTCATCCCTGCGTGTCTCCGACTCAG**CAGAAGGAA**CGATCAATGTCAACGACCGACC -3’	1678–1695
Barcode 6-A-CPRF1	5’- CCATCTCATCCCTGCGTGTCTCCGACTCAG**CTGCAAGTT**CGATCAATGTCAACGACCGACC -3’	1678–1695
Barcode 7-A-CPRF1	5’- CCATCTCATCCCTGCGTGTCTCCGACTCAG**TTCGTGATT**CGATCAATGTCAACGACCGACC -3’	1678–1695
Barcode 8-A-CPRF1	5’-CCATCTCATCCCTGCGTGTCTCCGACTCAG**TTCCGATAA**CGATCAATGTCAACGACCGACC -3’	1678–1695
Barcode 9-A-CPRF1	5’- CCATCTCATCCCTGCGTGTCTCCGACTCAG**TGAGCGGAA**CGATCAATGTCAACGACCGACC -3’	1678–1695
Barcode 10-A-CPRF1	5’- CCATCTCATCCCTGCGTGTCTCCGACTCAG**CTGACCGAA**CGATCAATGTCAACGACCGACC -3’	1678–1695
Barcode 11-A-CPRF1	5’- CCATCTCATCCCTGCGTGTCTCCGACTCAG**TCCTCGAAT**CGATCAATGTCAACGACCGACC -3’	1678–1695
Barcode 12-A-CPRF1	5’- CCATCTCATCCCTGCGTGTCTCCGACTCAG**TAGGTGGTT**CGATCAATGTCAACGACCGACC -3’	1678–1695
Barcode 13-A-CPRF1	5’- CCATCTCATCCCTGCGTGTCTCCGACTCAG**TCTAACGGA**CGATCAATGTCAACGACCGACC -3’	1678–1695
Barcode 14-A-CPRF1	5’- CCATCTCATCCCTGCGTGTCTCCGACTCAG**TTGGAGTGT**CGATCAATGTCAACGACCGACC -3’	1678–1695
Barcode 15-A-CPRF1	5’- CCATCTCATCCCTGCGTGTCTCCGACTCAG**TCTAGAGGT**CGATCAATGTCAACGACCGACC -3’	1678–1695
Barcode 16-A-CPRF1	5’- CCATCTCATCCCTGCGTGTCTCCGACTCAG**TCTGGATGA**CGATCAATGTCAACGACCGACC -3’	1678–1695
Barcode 17-A-CPRF1	5’- CCATCTCATCCCTGCGTGTCTCCGACTCAG**TCTATTCGT**CGATCAATGTCAACGACCGACC -3’	1678–1695
Barcode 18-A-CPRF1	5’- CCATCTCATCCCTGCGTGTCTCCGACTCAG**AGGCAATTG**CGATCAATGTCAACGACCGACC -3’	1678–1695
Barcode 19-A-CPRF1	5’- CCATCTCATCCCTGCGTGTCTCCGACTCAG**TTAGTCGGA**CGATCAATGTCAACGACCGACC -3’	1678–1695
Barcode 20-A-CPRF1	5’- CCATCTCATCCCTGCGTGTCTCCGACTCAG**CAGATCCAT**CGATCAATGTCAACGACCGACC -3’	1678–1695
Barcode 21-A-CPRF1	5’- CCATCTCATCCCTGCGTGTCTCCGACTCAG**TCGCAATTA**CGATCAATGTCAACGACCGACC -3’	1678–1695
Barcode 22-A-CPRF1	5’- CCATCTCATCCCTGCGTGTCTCCGACTCAG**TTCGAGACG**CGATCAATGTCAACGACCGACC -3’	1678–1695
Barcode 23-A-CPRF1	5’- CCATCTCATCCCTGCGTGTCTCCGACTCAG**TGCCACGAA**CGATCAATGTCAACGACCGACC -3’	1678–1695
Barcode 24-A-CPRF1	5’- CCATCTCATCCCTGCGTGTCTCCGACTCAG**AACCTCATT**CGATCAATGTCAACGACCGACC -3’	1678–1695
Barcode 25-A-CPRF1	5’- CCATCTCATCCCTGCGTGTCTCCGACTCAG**CCTGAGATA**CGATCAATGTCAACGACCGACC -3’	1678–1695
Barcode 26-A-CPRF1	5’- CCATCTCATCCCTGCGTGTCTCCGACTCAG**TTACAACCT**CGATCAATGTCAACGACCGACC -3’	1678–1695
TrP1- CPRR1	5’- CCTCTCTATGGGCAGTCGGTGAT GAGTAACTCCACAGTAGCTCC -3’	1928–1948

Table S2 Variants comparison of HBeAg positive CHB patients with that of HCC patients

Region	Variant	CHB (n=58) (%)	HCC (n=24)(%)	*P** value
EnhII	A1703G/C	7(12.1)	5(20.8)	0.3202
(1685-1773)	G1719T	28(48.3)	9(37.5)	0.4667
	G1721A	6(10.3)	8(33.3)	0.0213
	A1726C	18(31.0)	17(70.8)	0.0013
	A1727V[c] (T1727V[b])	30(51.7)	18(75.0)	0.0835
	C1730G[c] (G1730C[b])	8(13.8)	15(62.5)	< 0.0001
BCP	G1746A	56(96.6)	9(37.5)	< 0.0001
(1742-1849)	A1752G/T	17(29.3)	14(58.3)	0.0232
	T1753V	22(37.9)	12(50.0)	0.3351
	A1762T	35(60.3)	22(91.7)	< 0.0001
	G1764A	35(60.3)	21(87.5)	0.0192
	C1766T/G	6(10.3)	6(25.0)	0.1000
	T1768A/C	4(6.9)	9(37.5)	0.0014
	C1773T/A	4(6.9)	8(33.3)	0.0043
	A1775G/C	7(12.1)	6(25.0)	0.1861
	C1799G[c] (G1799C[b])	7(12.1)	16(66.7)	< 0.0001
	T1802C	5(8.6)	9(37.5)	0.0031
	T1803V	10(17.2)	7(29.2)	0.2433
PC	C1817G/A	18(31.0)	6(25.0)	0.7902
(1814-1900)	T1825C/A	58(100.0)	21(87.5)	0.0229
	A1846T/C	19(32.8)	15(62.5)	0.0156
	T1858C	5(8.6)	6(25.0)	0.0726
	G1896A	29(50.0)	18(75.0)	0.0501
	G1899A	10(17.2)	17(70.8)	< 0.0001

For all nucleotide positions in EnhII/BCP/PC regions, the most prevalent nucleotide type among HBV genotype B or genotype C infection patients in china were used as reference nucleotide. [c] Genotype C; [b] Genotype B; A, adenine; G, guanine; C, cytosine; T, thymine. Data presented as no. (%). *P**, Fisher's exact test.
